# Supplementary material for: A Dendritic Cell-Activating Rv1876 Protein Elicits Mycobacterium Bovis BCG-Prime Effect via Th1-Immune Response
Source: Biomolecules. 2021 Sep 3;11(9):1306. doi: 10.3390/biom11091306 (PMC8465531; doi:10.3390/biom11091306)
Supplement: Supplementary file 1 [file biomolecules-11-01306-s001.zip › Revised Supplementary Figure_biomolecules_1319244.pdf]

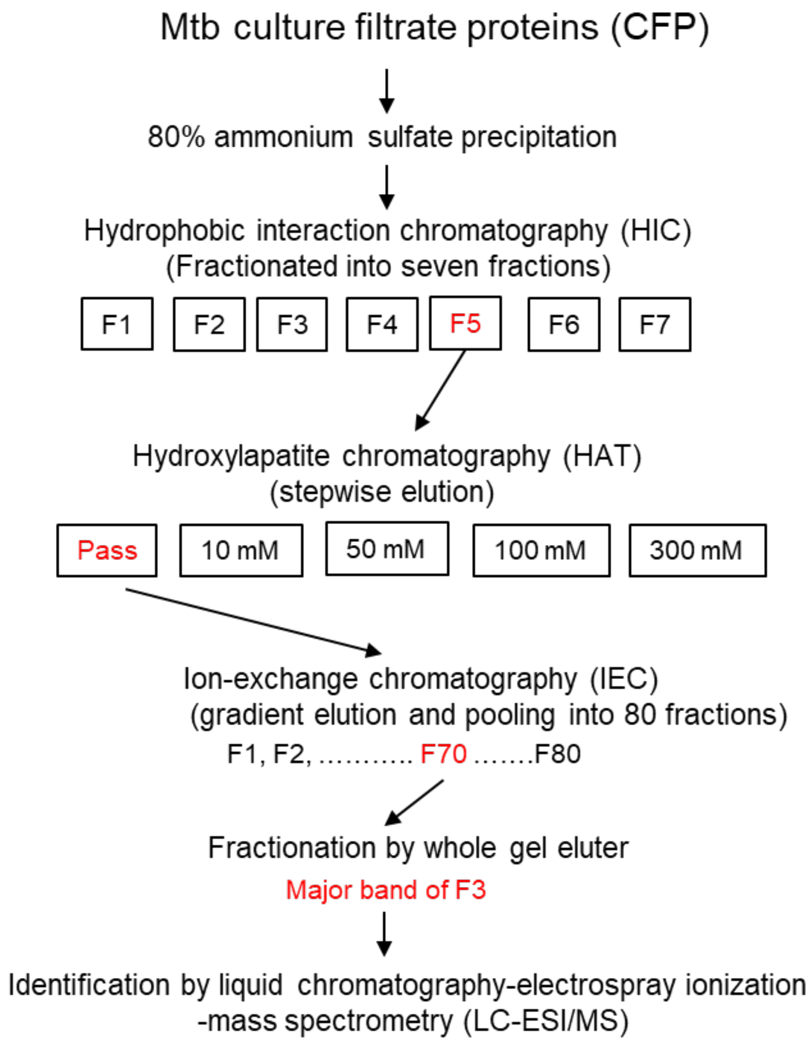

Supplementary Figure S1. Procedure of Multi-dimensional fractionation of Mtb CFP.

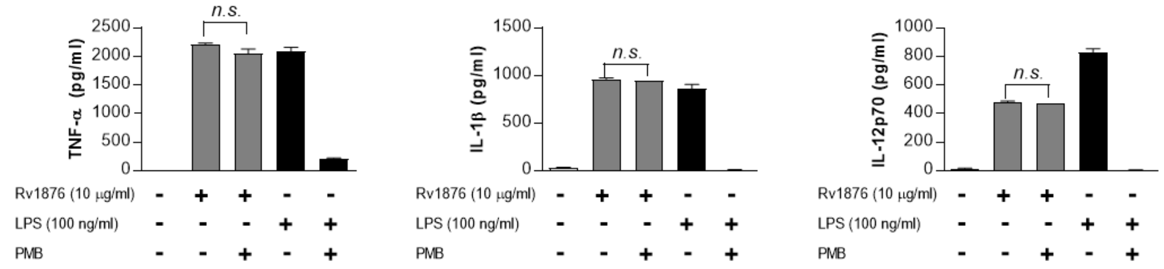

Supplementary Figure S2. Confirmation of endotoxin decontamination of the purified Rv1876. DCs were pretreated with polymyxin B (20 µg/mL) for 1 h prior to stimulation of Rv1876 (10 µg/mL). LPS (100 ng/mL) was used as control. After 24 h, the quantities of TNF-α, IL-1β, and IL-12p70 in the culture medium were measured by ELISAs. All data are expressed as mean ± SD (n = 3) and treatments that showed no significant effect are indicated as *n.s.*

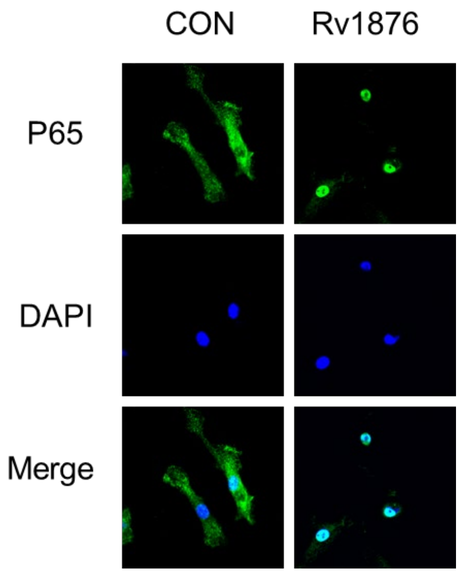

Supplementary Figure S3. Rv1876 enhances p65 nuclear translocation on BMDCs. The BMDC cells on coverslips were treated with Rv1876 for 45 min. Immunoreactivity of the p65 subunit of NF-κB was determined by immunofluorescence, as described in the Materials and Methods.

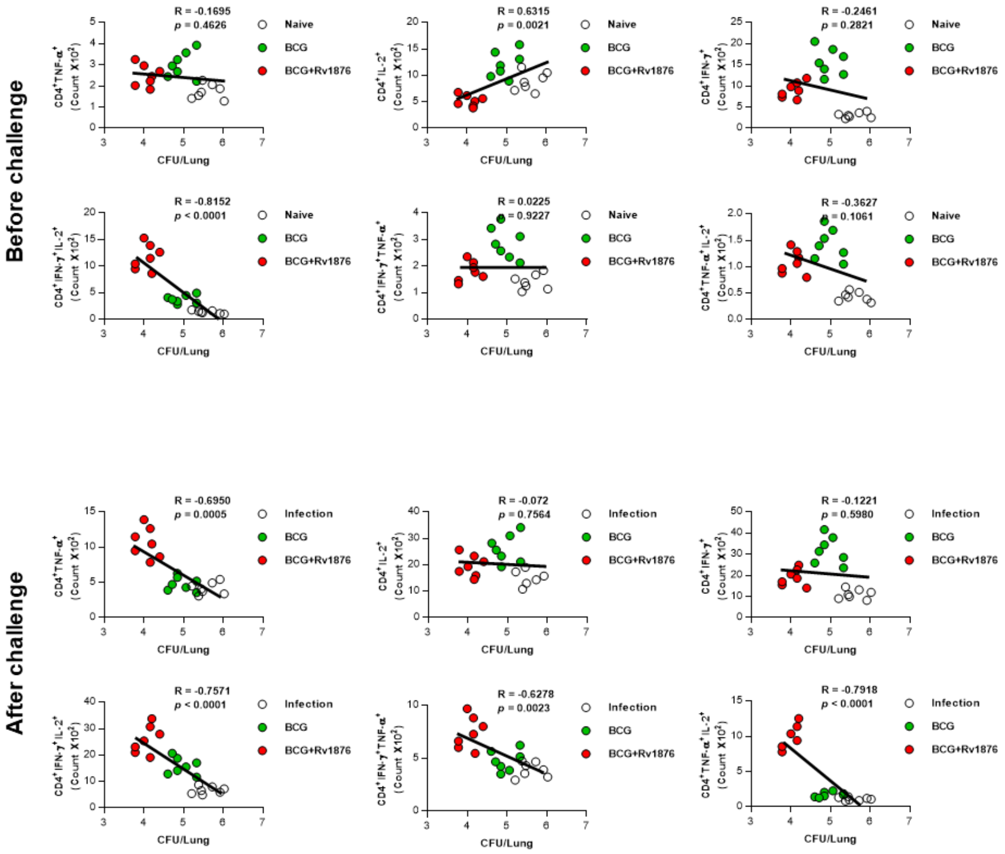

Supplementary Figure S4. Analysis of protective correlations for protection level with vaccine-induced immune responses before- and after-challenge. Relationship between protection (CFU) and Rv1876 specific various cytokine combination in $CD4^{+}CD44^{+}$ T cells is shown as a fitted regression line with the correlation coefficient. Spearman's r and P values of the correlations are indicated. White circle: Naïve or Infection, green circle: BCG, and red circle: BCG+Rv1876.
